# The mRNA-Binding Protein IGF2BP1 Restores Fetal Hemoglobin in Cultured Erythroid Cells from Patients with β-Hemoglobin Disorders

**DOI:** 10.1016/j.omtm.2020.01.011

**Published:** 2020-01-31

**Authors:** Christopher B. Chambers, Jeffrey Gross, Katherine Pratt, Xiang Guo, Colleen Byrnes, Y. Terry Lee, Donald Lavelle, Ann Dean, Jeffery L. Miller, Andrew Wilber

**Affiliations:** 1 Department of Medical Microbiology, Immunology and Cell Biology, Southern Illinois University School of Medicine, Springfield, IL 62702, USA; 2 Laboratory of Cellular and Developmental Biology, National Institute of Diabetes and Digestive and Kidney Diseases, National Institutes of Health, Bethesda, MD 20892, USA; 3 Genetics of Development and Diseases Branch, National Institute of Diabetes and Digestive and Kidney Diseases, National Institutes of Health, Bethesda, MD 20892, USA; 4 Section of Hematology/Oncology, Department of Medicine, University of Illinois at Chicago, Chicago, IL 60612, USA; 5 Jesse Brown VA Medical Center, Chicago, IL 60612, USA; 6 Simmons Cancer Institute, Springfield, IL 62702, USA

**Keywords:** fetal hemoglobin, beta-thalassemia, sickle cell disease, lentivirus, IGF2BP1, hemoglobinopathies, gene therapy, gene regulation

## Abstract

Sickle cell disease (SCD) and β-thalassemia are caused by structural abnormality or inadequate production of adult hemoglobin (HbA, α_2_β_2_), respectively. Individuals with either disorder are asymptomatic before birth because fetal hemoglobin (HbF, α_2_γ_2_) is unaffected. Thus, reversal of the switch from HbF to HbA could reduce or even prevent symptoms these disorders. In this study, we show that insulin-like growth factor 2 mRNA-binding protein 1 (IGF2BP1) is one factor that could accomplish this goal. IGF2BP1 is a fetal factor that undergoes a transcriptional switch consistent with the transition from HbF to HbA. Lentivirus delivery of IGF2BP1 to CD34^+^ cells of healthy adult donors reversed hemoglobin production toward the fetal type in culture-differentiated erythroid cells. Analogous studies using patient-derived CD34^+^ cells revealed that IGF2BP1-dependent HbF induction could ameliorate the chain imbalance in β-thalassemia or potently suppress expression of sickle β-globin in SCD. In all cases, fetal γ-globin mRNA increased and adult β-globin decreased due, in part, to formation of contacts between the locus control region (LCR) and γ-globin genes. We conclude that expression of IGF2BP1 in adult erythroid cells has the potential to maximize HbF expression in patients with severe β-hemoglobin disorders by reversing the developmental γ- to β-globin switch.

## Graphical Abstract

**Figure:**
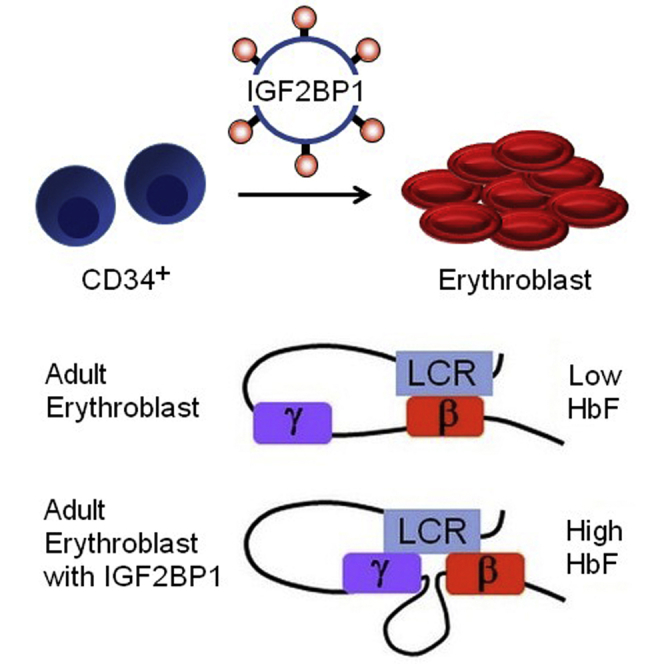

## Introduction

Red blood cells (RBCs) utilize hemoglobin to deliver oxygen from the lungs to cells and tissues. In humans, the composition of hemoglobin switches after birth when fetal hemoglobin (HbF, α_2_γ_2_) is replaced by adult hemoglobin (HbA, α_2_β_2_). This transition is pathologic for individuals who inherit mutations that cause sickle cell disease (SCD) or severe β-thalassemia. In SCD, the β-globin protein has a substitution of valine for glutamic acid that creates hemoglobin S (HbS, α_2_β^S^_2_).^1^ HbS forms rigid polymers when oxygen is released, causing RBCs to adopt a “sickle” shape. Sickle RBCs can aggregate and limit blood flow to tissues, resulting in severe pain, organ damage, and stroke.^1^ The β-thalassemias result from various alterations in the β-globin gene or its promoter that impact levels of HbA. Individuals with some level of β-globin protein are classified as β^+^, while those that lack expression have a β^0^ genotype. The severity of β-globin deficiency determines how much α-globin remains unpaired to form insoluble aggregates that cause RBC destruction and severe anemia, which is treated by frequent blood transfusions.^2^

Rare individuals with SCD or β-thalassemia also inherit genetic changes that cause HbF to be produced into adult life.^3^^,^^4^ These people have less severe disease or are asymptomatic depending on levels and distribution of HbF in RBCs.^4^ Medications that enhance HbF are being used and continue to be developed, but they are not a cure.^5^ Bone marrow (BM) transplantation from human leukocyte antigen (HLA)-matched siblings is a cure, but insufficient donors limit widespread use of this treatment.^6^^,^^7^ Alternative donor sources such as HLA-matched unrelated persons,^8^ HLA-mismatched family members,^9^ and umbilical cord blood units^10^ are being explored, but evidence is still needed to draw conclusive recommendations about safety and efficacy of these options. Thus, gene therapy using the patient’s own hematopoietic stem/progenitor cells (HSPCs, CD34^+^) creates an opportunity to increase availability of curative therapy while eliminating risks associated with heterologous transplant.^11^^,^^12^

Gene addition or modification strategies are being explored as treatment for β-hemoglobin disorders. Lentiviral vectors that encode for high-level, erythroid-specific expression of human γ-globin genomic sequences have been used by our group and others to improve or correct mouse models of β-thalassemia^13^^,^^14^ or SCD^15^^,^^16^ as well as erythroid progeny of CD34^+^ cells obtained from patients with these disorders.^17^^,^^18^ Other groups have demonstrated therapeutic benefit in similar mouse models^19^, ^20^, ^21^, ^22^, ^23^, ^24^ and cultured patient cells^25^, ^26^, ^27^, ^28^, ^29^, ^30^ using lentiviral vectors to express wild-type or modified versions of human β-globin. Clinical trials are ongoing for many of these approaches, with the most data available for lentiviruses expressing the human β-globin (β^T87Q^) with a beneficial γ-globin substitution.^31^, ^32^, ^33^ Interim results from these trials indicate that lentivirus delivery of β^T87Q^-globin gene is safe, well tolerated, and sufficient to achieve consistent clinical benefit in patients with less severe forms of transfusion-dependent β-thalassemia.

An alternative to globin gene addition is modulation of factors that regulate the endogenous fetal γ-globin (^G^γ and ^A^γ) genes. The transcription factors B cell CLL/lymphoma 11A (BCL11A) and leukemia/lymphoma-related factor (LRF) encoded by the ZBTB7A gene independently suppress γ-globin expression in adult erythroid cells,^34^, ^35^, ^36^, ^37^ and inhibition of either factor favors HbF production.^34^^,^^37^^,^^38^ Recently, insulin-like growth factor 2 mRNA binding protein 1 (IGF2BP1) was identified as a fetal-specific factor^39^^,^^40^ and positive regulator of HbF.^39^ IGF2BP1 is highly expressed during early development but suppressed in adult tissues; this timing pattern corresponds with the transition from HbF to HbA. Indeed, expression of IGF2BP1 in healthy adult erythroid cells caused a transcriptional switch from β- to γ-globin and robust increase in HbF.^39^ In this study, we confirm the ability of IGF2BP1 to potently induce HbF in adult erythroblasts and extend this function to cultured cells from patients with β-thalassemia major or SCD. In patient cells, IGF2BP1-dependent induction of HbF ameliorated the chain imbalance that occurs in β-thalassemia major or potently suppressed expression of sickle β-globin in SCD. Reversal of globin gene expression was achieved at the transcriptional level by increased γ-globin combined with decreased β-globin mRNA due, in part, to renewed interaction between the locus control region (LCR) and γ-globin genes. Based on these results, we propose that erythroid-specific expression of IGF2BP1 could provide dominant and curative levels of HbF in RBCs derived from transduced HSPCs of patients with SCD or severe β-thalassemia.

## Results

### IGF2BP1 Is Highly Expressed in Human Fetal Erythroblasts but Silenced in Adult Erythroblasts

**Figure 1:**
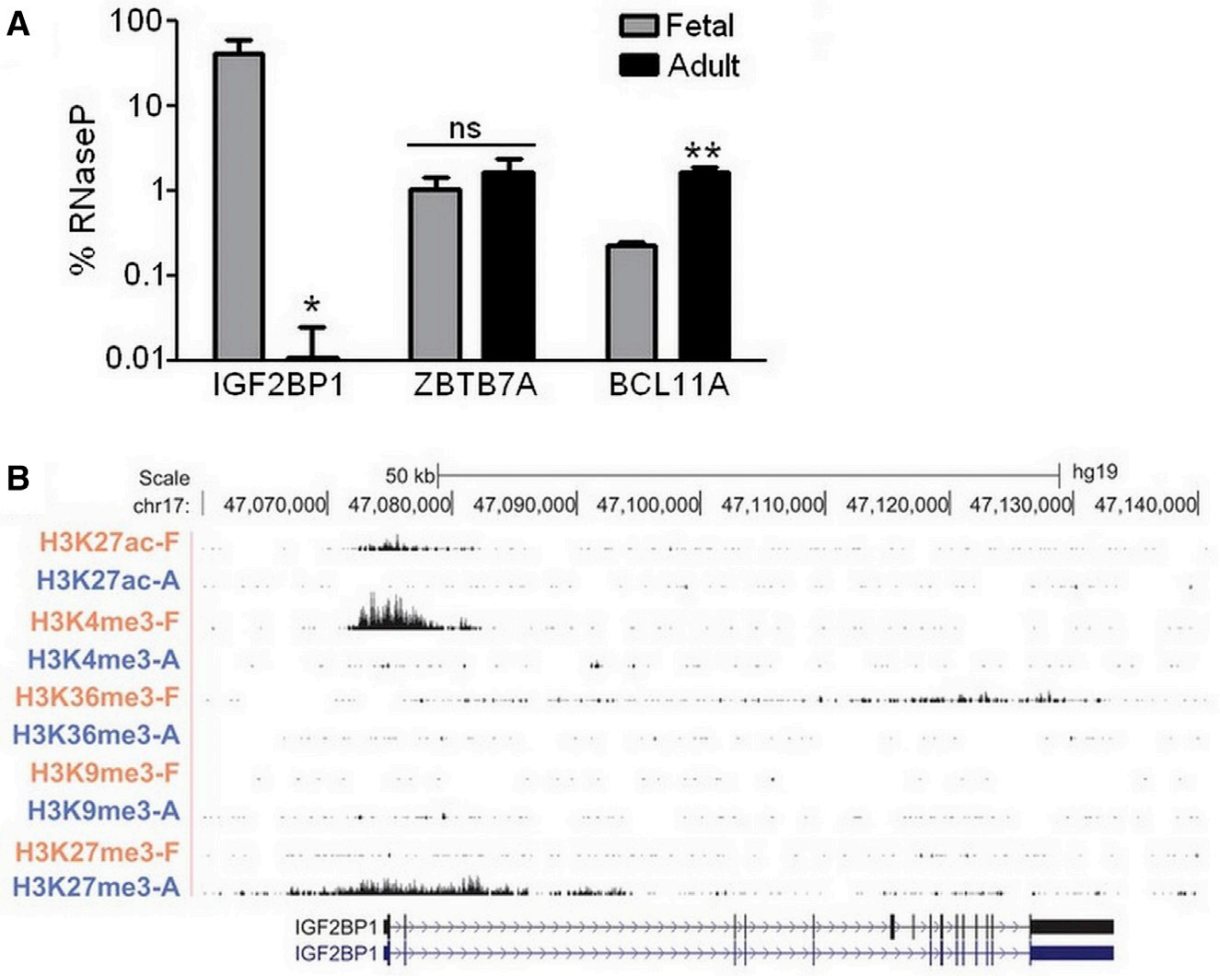
Expression and Epigenetic Analysis of IGF2BP1 in Fetal and Adult Erythroblasts (A) qRT-PCR analysis of IGF2BP1, LRF/ZBTB7A, and BCL11A mRNA in erythroblasts derived from fetal liver versus adult bone marrow CD34^+^ cells after 8 days of culture (n = 3 donors each). The expression level is reported as percentage of the internal control RNaseP and the mean ± SD is plotted. (B) ChIP sequencing results used to generate a genome-wide map of histone modifications in culture-differentiated fetal and adult erythroblasts^37^ were re-analyzed to determine epigenetic modifications for IGF2BP1. Shown are results for markers of active chromatin: H3K27 acetylation (H3K27ac), H3K4 trimethylation (H3K4me3), and H3K36 trimethylation (H3K36me3) or repressive chromatin: H3K9 trimethylation (H3K9me3) and K3K27 trimethylation (H3K27me3) in fetal (orange) and adult cells (blue). ns, not significant; *p ≤ 0.01, **p ≤ 0.001 determined by unpaired Student’s t test (two-tailed).

The beneficial effects of HbF on the severity of β-thalassemia and SCD have fueled long-standing efforts to identify factors controlling expression of the endogenous fetal γ-globin genes. These studies have identified numerous gene products that can be modulated to induce HbF expression,^41^ including IGF2BP1.^39^ Association between IGF2BP1 and developmental hemoglobin switching was initially revealed by comparing RNA and protein expression for reticulocytes and culture-differentiated erythroid cells that predominantly produced HbF versus those that did not.^39^ To confirm fetal-specific expression of IGF2BP1, we used our two-phase culture model of human erythropoiesis.^42^ In this system, CD34^+^ cells are first proliferated and then differentiated for 7 days each. Terminal stage cultures consist of mostly orthochromatic erythroblasts, and hemoglobin production mirrors the developmental state of the starting cell population. Specifically, CD34^+^ cells isolated from fetal liver (FL) produce predominantly HbF while those from adult BM produce mostly HbA.^42^ Erythroblasts were generated from FL CD34^+^ cells purchased from Lonza (Walkersville, MD, USA) or adult BM CD34^+^ cells obtained from either Lonza or the Yale Cooperative Center of Excellence in Hematology (Yale School of Medicine, New Haven, CT, USA). qRT-PCR was used to quantify transcript levels of IGF2BP1 as a percentage of the internal control RNaseP (Figure 1A). This analysis also included primer-probe sets capable of detecting all BCL11A splice variants or ZBTB7A, two well-known transcriptional repressors of the fetal γ-globin genes.^34^, ^35^, ^36^, ^37^ IGF2BP1 was highly expressed in fetal erythroblasts (42 ± 19% of RNaseP), but at background levels in adult counterparts (0.01% ± 0.01%), a 4,200-fold change. Alternatively, BCL11A transcripts were modestly increased in adult cells (0.2% ± 0.02% fetal versus 1.6% ± 0.3% adult), and LRF/ZBTB7A was expressed at similar levels in both groups (1.0% ± 0.4% fetal versus 1.7% ± 0.8% adult).

A prior study used culture-differentiated human fetal and adult erythroblasts and the chromatin immunoprecipitation (ChIP)-sequencing method to develop a genome-wide map of histone modifications and make comparisons across the β-globin locus.^43^ Consistent with differential globin gene expression for these developmental stages, epigenetic modifications typical of active chromatin regions (H3K27ac, H3K4me3, and H3K36me3) were enriched at the ^G^γ- and ^A^γ-globin genes in fetal cells and the β-globin gene in adult cells. Conversely, repressive chromatin markers (H3K9me3 and H3K27me3) were detected at the γ-globin genes in adult cells and β-globin in fetal cells. We re-analyzed these data to define the chromatin pattern for IGF2BP1, BCL11A, and LRF/ZBTB7A for fetal and adult erythroblasts. For IGF2BP1, active chromatin modifications were observed in fetal cells and the repressive marks limited to adult cells (Figure 1B). Consistent with these marks, flow cytometry analysis of culture-differentiated erythroblasts confirmed that IGF2BP1 was expressed in fetal cells, which produce HbF, versus adult cells, which do not (Figure S1). Conversely, BCL11A and LRF/ZBTB7A exhibited active chromatin marks in both cell types, and these signals were enriched in adult cells (Figures S2A and S2B). From these data, we conclude that IGF2BP1 expression is specific to fetal erythroid development and undergoes a transcriptional switch that correlates with the transition from HbF to HbA production.

### IGF2BP1 Reverses Hemoglobin Expression in Healthy Adult Erythroblasts

Previously, it was shown that lentivirus-mediated expression of IGF2BP1 in culture-differentiated erythroblasts of healthy adult donors could reverse hemoglobin production to the fetal type.^39^ This was accomplished with a lentiviral vector that utilized a relatively weak human spectrin alpha gene (SPTA1) promoter to drive erythroid-specific expression of IGF2BP1 and a puromycin resistance gene coupled by an internal ribosomal entry site (IRES) element (SIiP, Figure S3A). We replaced the IRES with the 2A self-cleaving peptide from porcine teschovirus-1 (P2A) to reduce the size of the lentiviral vector cargo as well as provide efficient cleavage between IGF2BP1 and downstream marker genes.^44^ The 2A sequence was linked to a puromycin resistance gene or ZsGreen fluorescent reporter^45^ to permit drug selection or visual confirmation and enrichment of transduced cells by fluorescence-activated cell sorting (FACS), respectively (Figure S3A). To determine the impact of these modifications on IGF2BP1 function, we transduced CD34^+^ cells from four healthy adult donors with the parental SIiP virus or versions that included the 2A-puromcyin (SI2AP) or 2A-ZsGreen (SI2AZG) cassette at equivalent multiplicities of infection (MOIs) (Figure S3B). Control cells were transduced using mock conditions or with an MSCV-regulated GFP virus, which we have shown to provide high levels of gene transfer in human CD34^+^ cells and subsequent expression in erythroid progeny.^17^^,^^42^ Transduced cells were grown for 2 days before the IGF2BP1 fraction was enriched by FACS for ZsGreen or selected with puromycin. After 5 days of additional growth, cells were collected to obtain total RNA and protein, perform flow cytometry analysis, or for plating into differentiation medium (Figure S3B).

**Figure 2:**
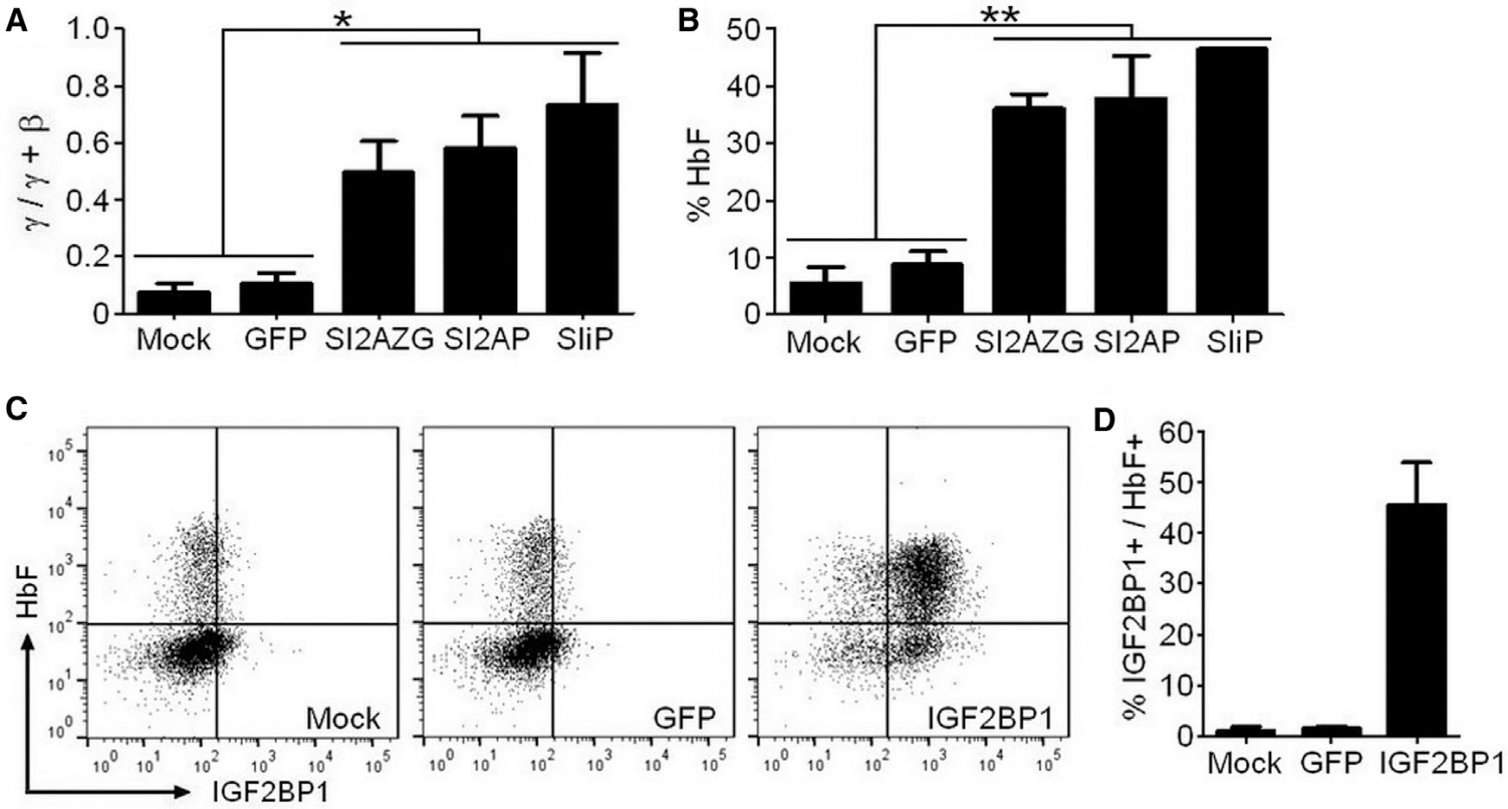
IGF2BP1 Induces a Fetal Pattern of Hemoglobin Expression in Healthy Adult Erythroblasts CD34^+^ cells from four healthy adult donors were transduced using mock conditions or with lentivirus encoding for GFP (control) or an α-spectrin regulated IGF2BP1 cassette that included 2A-ZsGreen (SI2AZG), 2A-puromycin (SI2AP), or IRES-puromycin (SIiP) and differentiated into erythroblasts. (A) qRT-PCR analysis showing the ratio of γ-globin mRNA to total (γ-globin + β-globin) mRNA plotted as mean ± SEM. (B) Percentage HbF of total hemoglobin (HbF + HbA) determined by HPLC of differentiated cell lysates for the indicated conditions plotted as mean ± SEM. (C) Representative dot plots showing expression of IGF2BP1 and HbF for mock, GFP, or IGF2BP1-transduced cells. (D) Percentage of cells double positive for IGF2BP1 and HbF plotted as mean ± SEM. *p ≤ 0.05, **p ≤ 0.001 determined by one-way ANOVA with Newman-Keuls *post hoc* analysis.

As previously observed,^39^ SIiP vector-transduced cells overexpressing IGF2BP1 favored production of HbF. Molecular analysis confirmed that SIiP-mediated HbF production resulted from a significant shift in the ratio of γ-globin/total (γ- + β-globin) mRNA compared to mock and GFP controls (Table 1; Figure 2A). Importantly, our modified SPTA1-IGF2BP1-2A vectors (SI2AP and SI2AZG) yielded similar increases in γ-globin over total β-like globin mRNA (Table 1; Figure 2A). High-performance liquid chromatography (HPLC) analysis of lysates from differentiated cells confirmed that IGF2BP1-mediated effects on globin transcription coincided with high levels of HbF (HbF/[HbF + HbA]) protein (Table 1; Figure 2B). To verify that HbF induction was coordinated with vector-mediated expression of IGF2BP1, we performed flow cytometry on erythroblasts that had been permeabilized and incubated with antibodies to IGF2BP1 and HbF. Minor cell populations with low levels of HbF or IGF2BP1 were identified for mock and GFP control cells, and only a few percent co-expressed both proteins (Figures 2C and 2D; % IGF2BP1^+^/HbF^+^, mean ± SEM: mock, 1.1 ± 0.7; GFP, 1.5 ± 0.4). Alternatively, IGF2BP1-transduced cells exhibited high levels of both IGF2BP1 and HbF that was predominantly coordinated (Figures 2C and 2D; % IGF2BP1^+^/HbF^+^, mean ± SEM: IGF2BP1, 45.6 ± 8.3). Thus, expression of IGF2BP1 in adult erythroblasts can potently reverse hemoglobin production toward a fetal-like phenotype, and this function is independent of the marker gene used to identify and/or enrich the transduced cell populations.

**Table 1:** Increased Levels of γ-Globin and HbF in Erythroblasts Derived from Adult CD34^+^ Cells Transduced with IGF2BP1 Lentiviral Vectors

Transduction	γ-Globin mRNA^a^	% HbF^b^
Mock	0.06 ± 0.02	4.5 ± 2.4
GFP	0.10 ± 0.04	6.3 ± 1.8
SIiP	0.57 ± 0.20	46.5 ± 0.10
SI2AP	0.52 ± 0.10	33.1 ± 5.5
SI2AZG	0.50 ± 0.11	36.1 ± 2.5

Data are mean ± SEM.

a Ratio of γ-globin to total (γ-globin + β-globin) mRNA was determined by qRT-PCR.

b Percentage of HbF to total (HbF + HbA) was determined by HPLC.

### IGF2BP1 Augments HbF in β-Thalassemia Major Erythroblasts

Having established function of the newly constructed 2A vectors in healthy erythroblasts, we wanted to determine whether IGF2BP1 could induce HbF levels in cultured cells from patients with hemoglobin disorders. This was first tested using CD34^+^ cells isolated from BM of two patients with β-thalassemia, which we have shown to have suboptimal levels of HbA when differentiated in culture.^17^ Cells were mock treated or transduced with a lentivirus encoding for the GFP control or the IGF2BP1-2A-puromycin cassette under control of the erythroid-specific SPTA1 promoter (SI2AP) or the constitutive SFFV promoter (FI2AP). Transduced cells were expanded for 2 days and then cultured for 5 more days in a low concentration of puromycin to enrich the IGF2BP1 fraction. At this time point, cells were collected for isolation of total RNA, protein, or flow cytometry analysis and the remainder were plated into differentiation medium.

**Figure 3:**
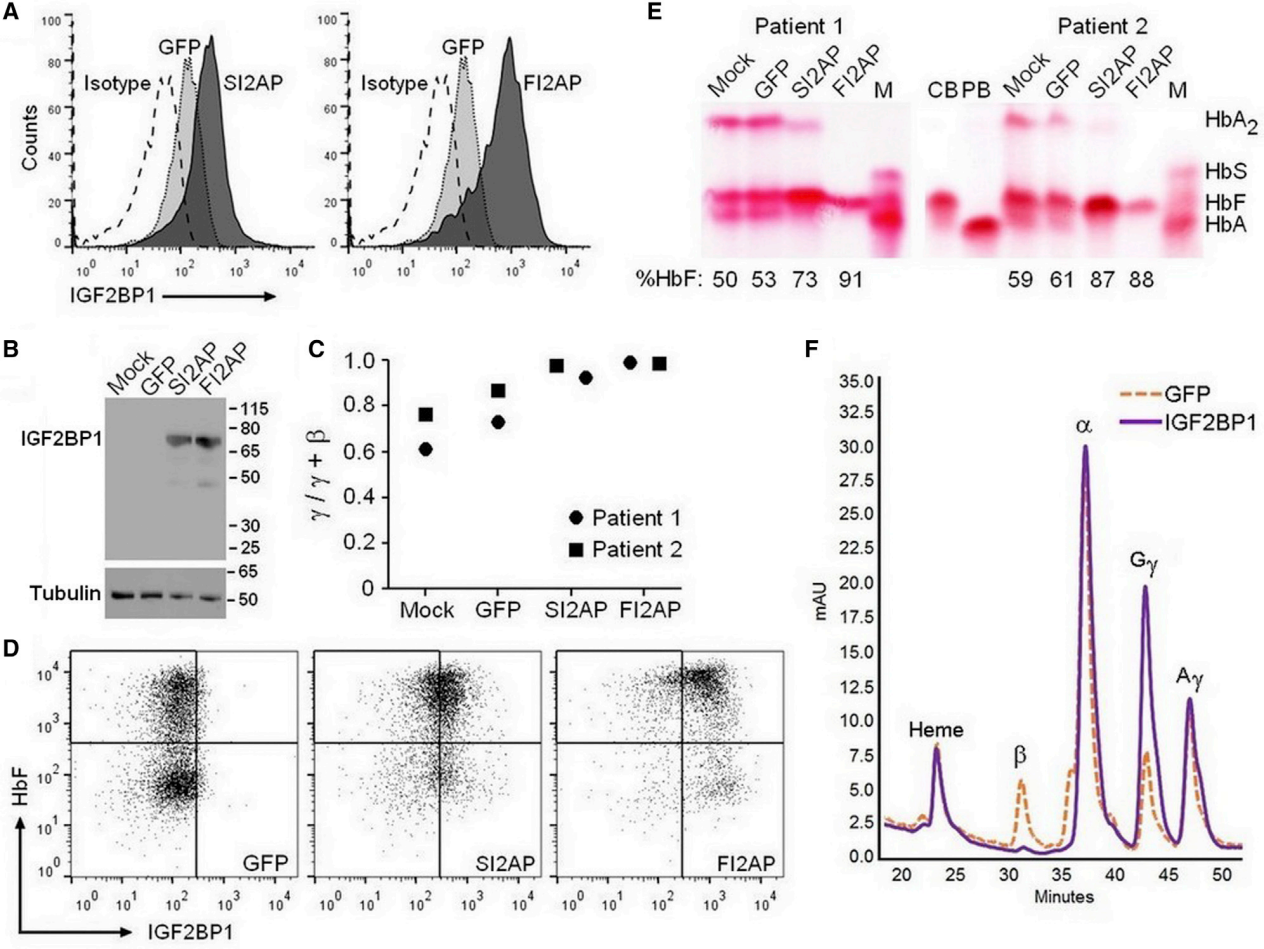
IGF2BP1 Augments HbF in β-Thalassemia Erythroblasts Bone marrow CD34^+^ cells from two adults diagnosed with β-thalassemia were transduced using mock conditions or with lentivirus encoding for GFP (control) or the IGF2BP1-2A-puromycin cassette under control of the erythroid-specific SPTA1 promoter (SI2AP) or constitutive SFFV promoter (FI2AP). Transduced cells were expanded for 7 days before samples were collected for RNA, protein, or flow cytometry and the remaining cells were placed into differentiation medium. (A) Overlay of representative flow cytometry histograms and (B) western blot demonstrating expression of IGF2BP1 for the indicated transduction conditions. Tubulin signal, shown below each lane of the western blot, served as a loading control. Molecular mass is shown in kDa. (C) qRT-PCR analysis showing the ratio of γ-globin mRNA to total (γ-globin + β-globin) mRNA for each patient. (D) Dot plots showing expression of IGF2BP1 and HbF for cells transduced with GFP control or IGF2BP1 under control of the SPTA1 (SI2AP) or SFFV (FI2AP) promoter. (E) Cellulose acetate hemoglobin electrophoresis of lysates from differentiated erythroblasts. Control samples included lysates prepared from peripheral blood of a healthy donor (PB) or umbilical cord blood (CB). M, standard consisting of sickle (HbS), fetal (HbF), and adult (HbA) hemoglobin. Percentage HbF of total hemoglobin (HbF + HbA-like [HbA_2_, HbA]) determined by HPLC of differentiated cell lysates is reported below each lane. (F) Reverse-phase HPLC analysis of globin chains in differentiated cell lysates for GFP control (orange) and IGF2BP1 (purple) cell populations.

Control β-thalassemia cells (mock and GFP) had very low levels of IGF2BP1 mRNA, which were greatly elevated for IGF2BP1-transduced cells (Table 2). Flow cytometry for IGF2BP1 protein correlated with qRT-PCR results, as only a small percentage of positive cells was detected in control populations, which was greatly increased in IGF2BP1 transduced populations (Table 2; Figure 3A). Low levels of IGF2BP1 protein detected in mock and GFP control cells by flow cytometry were not appreciated by western blot of samples from the same time point (Figure 3B). Compared to healthy donors, β-thalassemia erythroblasts had a higher basal level of γ-globin to total β-like globin mRNA (compare mock and GFP in Tables 1 and 2). Still, IGF2BP1 shifted the total β-like globin mRNA profile to almost entirely γ-globin (Table 2; Figure 3C), resulting in pancellular distribution of HbF by flow cytometry (Figure 3D). Cellulose acetate hemoglobin electrophoresis and HPLC analysis of cell lysates showed that mock and GFP vector-transduced β-thalassemia cells produced HbF and some HbA and HbA_2_ (α_2_δ_2_), which is consistent with these patients having a β^+^/β^0^ or β^+^/β^+^ thalassemia genotype.^17^^,^^46^ IGF2BP1 augmented levels of HbF and simultaneously decreased both HbA and HbA_2_ (Table 2; Figure 3E), indicating a global effect on adult hemoglobin gene expression and reversal of the γ-to-β switch even though these patients had deficient β-globin expression. This point is reinforced by enrichment of ^G^γ-globin and reduction in β-globin for IGF2BP1-expressing cells versus controls when lysates were subjected to reverse-phase HPLC (Figure 3F).

**Table 2:** Increased Levels of γ-Globin and HbF in Erythroblasts Derived from Bone Marrow CD34^+^ Cells of β-Hemoglobinopathy Patients Transduced with IGF2BP1 Lentiviral Vectors

	β-Thalassemia Patient 1				β-Thalassemia Patient 2				SCD Patient			
	Mock	GFP	SI2AP	FI2AP	Mock	GFP	SI2AP	FI2AP	Mock	GFP	SI2AP	FI2AP
IGF2BP1 mRNA^a^	1.4	0.7	8.6	101.8	0.03	0.03	17.3	65.8	0.18	0.06	46	141
% IGF2BP1^+^^b^	8	9	59	79	8	13	52	60	32	37	70	74
γ-Globin mRNA^c^	61.1	73.1	92.3	99.2	76.5	86.6	97.6	98.8	33.0	56.8	91.2	96.5
% HbA or HbS^d^	27	24	19	9	26	27	9	12	81	74	33	23
% HbA_2_^d^	23	23	8	0	15	12	4	0	4	5	0	0
% HbF^d^	50	53	73	91	59	61	87	88	15	21	67	77

a IGF2BP1 mRNA as percentage of RNaseP determined by qRT-PCR.

b Percentage of IGF2BP1-positive cells determined by flow cytometry.

c Ratio of γ-globin to total β-like globin (γ-globin + β-globin) mRNA determined by qRT-PCR.

d Percentage to total hemoglobin (HbF + HbA/S + HbA_2_) determined by HPLC.

### IGF2BP1 Suppresses β^S^-Globin in SCD Erythroblasts

**Figure 4:**
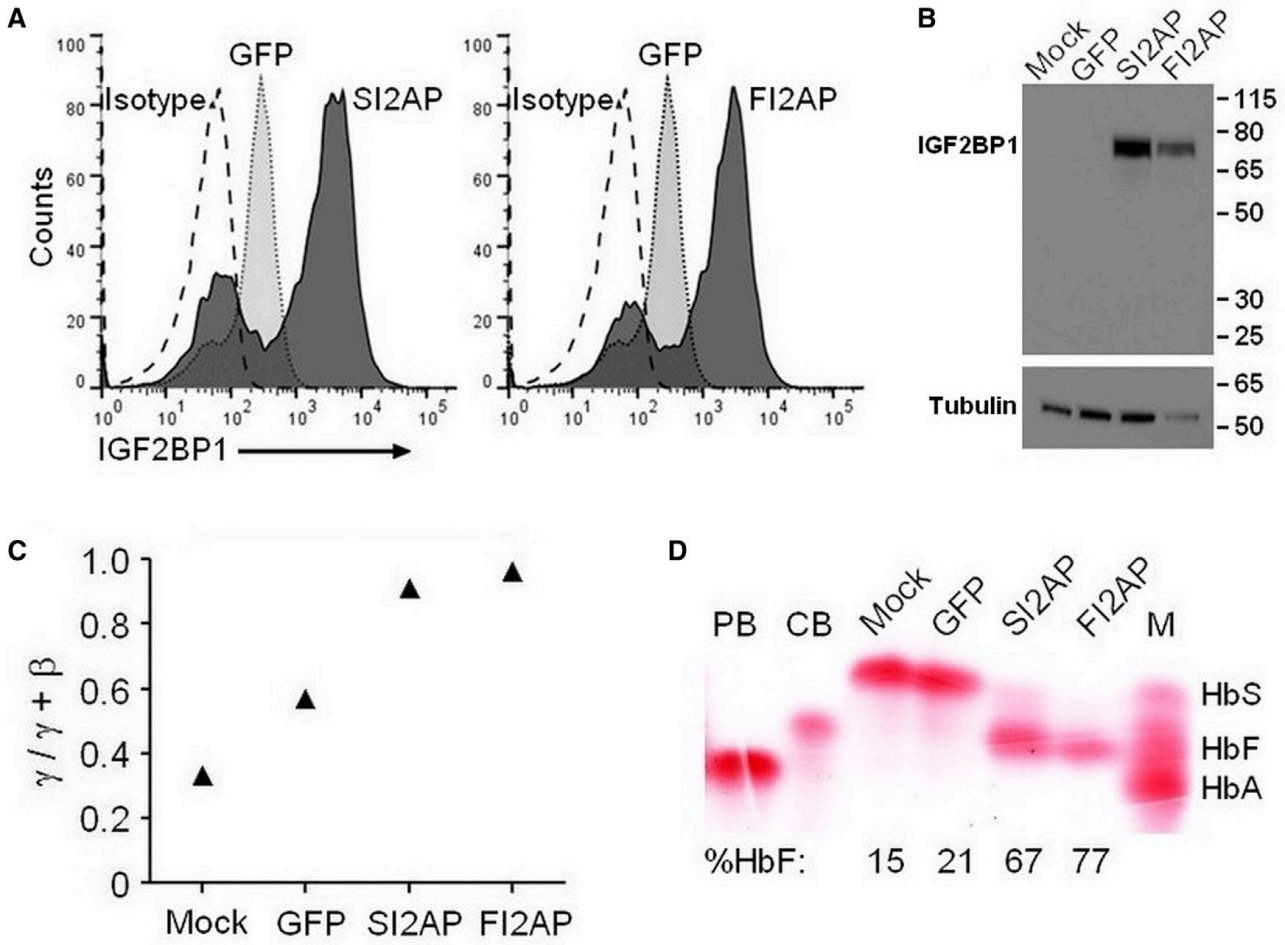
IGF2BP1 Reverses Defective β-Globin Expression in SCD Erythroblasts Bone marrow CD34^+^ cells from an SCD patient were transduced using conditions identical to those for the β-thalassemia patients and equivalent samples were collected. (A and B) Expression of IGF2BP1 for the indicated transduction conditions demonstrated by (A) overlay of flow cytometry histograms and (B) western blot. Tubulin signal, shown below each lane of the western blot, served as a loading control. Molecular mass is shown in kDa. (C) qRT-PCR analysis showing the ratio of γ-globin mRNA to total (γ-globin + β-globin) mRNA. (D) Cellulose acetate hemoglobin electrophoresis of lysates from differentiated erythroblasts. Control lysates were from peripheral blood of a healthy donor (PB) or umbilical cord blood (CB). M, standard consisting of sickle (HbS), fetal (HbF), and adult (HbA) hemoglobin. Percentage HbF of total hemoglobin (HbF + HbA-like [HbA_2_, HbA, HbS]) determined by HPLC of differentiated cell lysates is reported below each lane.

Our results for healthy donors and β-thalassemia patients demonstrate that IGF2BP1 can reverse the developmental hemoglobin switch. We sought to determine the impact of IGF2BP1 on HbF and HbS levels in SCD patient-derived erythroblasts using identical transduction and selection conditions. Again, IGF2BP1 mRNA was very low to undetectable in control samples and substantially increased in cells transduced with SI2AP and FI2AP vectors (Table 2). IGF2BP1 protein was detected in mock and GFP control cells by flow cytometry, but percentages were greatly increased for transduced populations (Table 2; Figure 4A). These levels of IGF2BP1 protein were only visible by western blot for the SI2AP and FI2AP samples (Figure 4B), which mirrors results for β-thalassemia cells. The basal level of γ-globin mRNA in controls (mock and GFP) was lower than that seen in β-thalassemia cells, likely due to the fact that SCD cells express wild-type levels of β^S^-globin. The level of γ-globin rose in response to IGF2BP1 whereas β-globin was reduced, a shift that almost exclusively favored γ-globin (Table 2; Figure 4C). Cellulose acetate electrophoresis (Figure 4D) and HPLC (Figure S4) confirmed the transcriptional switch with IGF2BP1-transduced cells predominantly producing HbF and mock and GFP controls producing mainly HbS (Table 2; Figure 4D). Thus, IGF2BP1-mediated effects may be sufficient to provide dominant and potentially curative levels of HbF in SCD patients, but this will require additional patient samples to confirm.

### IGF2BP1 Induces γ-Globin/LCR Looping

**Figure 5:**
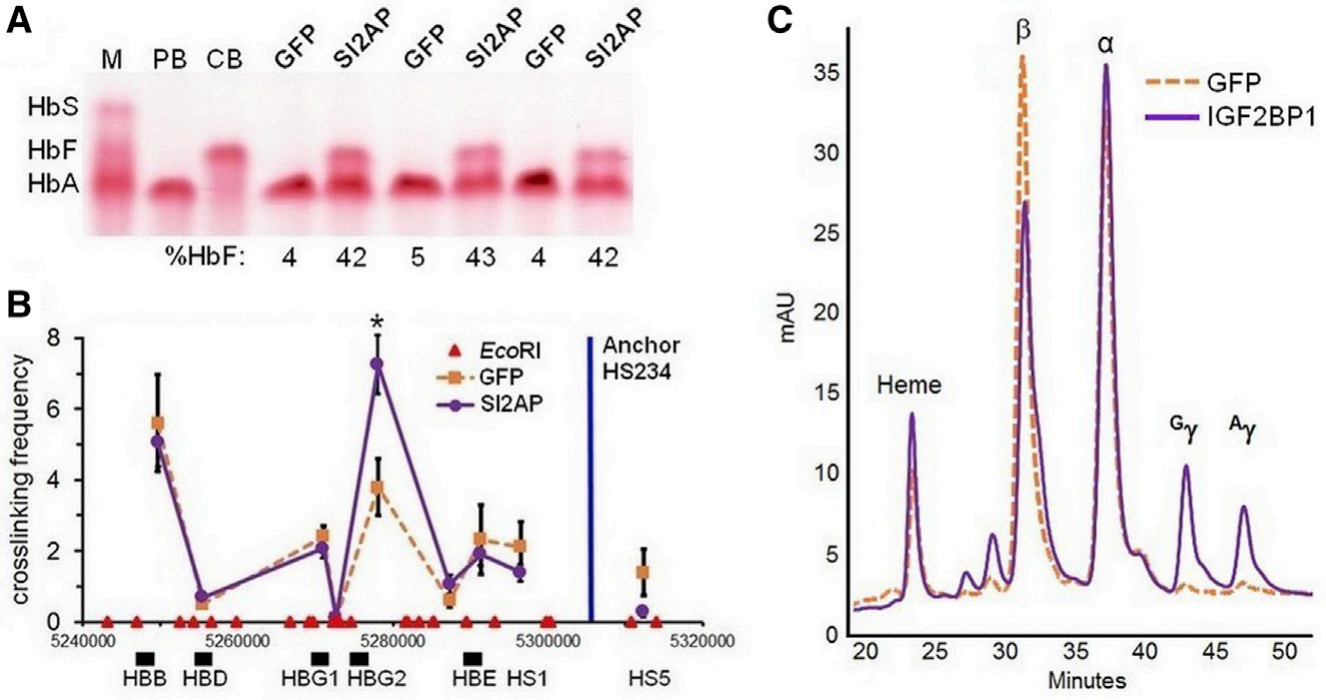
IGF2BP1 Promotes Interaction between the LCR and γ-Globin Genes Cytokine-mobilized CD34^+^ cells from a healthy adult donor were transduced in triplicate with GFP control or SI2AP lentivirus particles. (A) Hemoglobin electrophoresis of differentiated cell lysates. Percentage HbF of total hemoglobin (HbF + HbA) determined by HPLC is reported below each lane. Control samples were from adult peripheral blood (PB) or umbilical cord blood (CB). M, standard consisting of sickle (HbS), fetal (HbF), and adult (HbA) hemoglobin. (B) 3C assay measuring relative crosslinking frequencies between the anchor fragment (vertical blue line) and globin genes in GFP control (orange) and IGF2BP1 (purple)-expressing cells. Each *Eco*RI cleavage site is represented by a red triangle, and globin genes are identified with black rectangles. Data are plotted as mean ± SD. (C) Reverse-phase HPLC analysis of globin chains in differentiated cell lysates for GFP control (orange) and IGF2BP1 (purple) cell populations. *p ≤ 0.05 determined by unpaired Student’s t test (two-tailed).

The LCR is an enhancer that physically interacts with the globin gene promoters to drive high-level, erythroid-specific expression during development. In fetal erythroblasts, the LCR binds upstream of the ^A^γ- and ^G^γ-globin genes, leading to HbF production, whereas in adult cells, the LCR localizes with the β-globin gene promoter to confer HbA expression.^3^^,^^47^ To determine whether IGF2BP1 expression would cause the LCR to re-establish connections with the γ-globin genes, a chromosome conformation capture (3C) experiment was performed to assess the relative interaction of the LCR with the γ- or β-globin genes in adult erythroblasts. CD34^+^ cells from a healthy adult donor were transduced in triplicate with either GFP control or SI2AP lentivirus particles. Transduced cells were grown for 2 days and then selected with puromycin to enrich the IGF2BP1 population. After 5 days of expansion, samples were equally divided and fixed with formaldehyde to stabilize DNA/protein interactions or transferred to differentiation medium. Hemoglobin electrophoresis showed uniform induction of HbF (mean of 42% by HPLC) for samples expressing IGF2BP1 (Figure 5A). Consistent with other studies,^44^, ^45^, ^46^, ^47^, ^48^, ^49^, ^50^ control cells demonstrated interaction between the LCR with β-globin gene (Figure 5B). IGF2BP1 significantly increased the interaction frequency of the LCR with the γ-globin gene (Figure 5B). The experiment was repeated using the FI2AP lentivirus and GFP control. Mean levels of HbF (66% by HPLC) surpassed those achieved using the SI2AP lentivirus (Figure S5A). Relative interaction of the LCR with the γ-globin genes was, however, not further augmented (Figure S5B). These data demonstrate that IGF2BP1 acts to partially re-localize the LCR to allow expression of the γ-globin genes in adult erythroblasts, which was confirmed by increased expression of both ^G^γ- and ^A^γ-globin proteins compared with GFP control when lysates from differentiated cells were analyzed by reverse-phase HPLC (Figure 5C).

## Discussion

Our data confirm previous findings that IGF2BP1 is expressed at high levels in fetal erythroblasts and at very low to undetectable levels in adult erythroblasts.^39^^,^^40^ This is not unexpected, as multicellular organisms have networks of temporally regulated genes responsible for controlling major developmental events, including those that occur during the transition from fetal to adult life.^51^ The IGF2BP family consists of three proteins (IGF2BP1, 2, and 3). All three encode two RNA recognition motifs and four heterogeneous nuclear ribonucleoprotein (hnRNP) K homology domains.^52^ IGF2BP2 is the only member that continues to be expressed in adult life, while the other two IGF2BP genes are downregulated as tissues mature.^53^ IGF2BP1 has well-defined roles in controlling the stability, localization, and translation of RNA targets, such as IGF2, β-actin, tau, CD44, and β-catenin.^54^ These functions are essential for normal growth and development, as mice deficient in *Igf2bp1* have dwarfism and impaired intestinal development.^53^ The presence of IGF2BP1 in some tumors has led to the suggestion that it is oncogenic. Activation of IGF2BP1 in cancer could result from reduced expression of *let-7* microRNAs, which regulate thousands of transcripts, including the IGF2BPs.^55^ Nevertheless, oncogenic potential is cell- and tissue type-dependent, as IGF2BP1 also can act to suppress proliferation and invasiveness of metastatic cells.^56^ This diversity of effects may be due to the large predicted number of potential IGF2BP1 target RNAs in cell lines^57^ and primary cells.^58^ Genome-wide association studies (GWASs) have not identified any potential IGF2BP1 single-nucleotide polymorphisms (SNPs) linked to human disease whereas SNPs in the second intron of IGF2BP2 are associated with type 2 diabetes.^59^^,^^60^

The best characterized of the γ-globin regulators is the zinc-finger transcription factor BCL11A. We found that BCL11A is expressed in fetal erythroblasts and upregulated in adult counterparts, which is consistent with its role as a suppressor of the γ-globin genes.^34^, ^35^, ^36^^,^^38^ Analysis of published ChIP-sequencing data for fetal and adult erythroblasts^43^ complement this finding, as the *BCL11A* locus exhibited active chromatin marks in both cell populations that were enhanced in adult cells. Conversely, *IGF2BP1* was marked by histone modifications typical of repressed chromatin in adult cells. Indeed, IGF2BP1 protein was significantly lower for differentiated erythroblasts from BM CD34^+^ cells compared with FL counterparts. Lentivirus-mediated overexpression of IGF2BP1 in differentiated erythroblasts of healthy adult donors and patients with β-hemoglobin disorders reversed the pattern of globin gene expression to favor the fetal type. Reverse-phase HPLC analysis of globin chains revealed a preferential increase in ^G^γ- versus ^A^γ-globin for β-thalassemia cells (Figure 3F) that was not apparent for healthy donors (Figure 5C). This deferential effect on globin gene expression is unexpected and will require further experimentation to explain.

A previous study established that IGF2BP1 overexpression in adult erythroblasts reduced levels of BCL11A protein.^39^ This effect was considered to occur post-transcriptionally, as IGF2BP1 overexpression had no effect on BCL11A mRNA level or stability.^39^ Sequence analysis of RNA that immunoprecipitated with IGF2BP1 identified BCL11A. Quantification of BCL11A transcripts in polysome fractions revealed no difference for control and IGF2BP1-expressing cells.^39^ This result could be explained by findings that IGF2BP1-containing RNP granules lack factors (eIF4E, eIF4G, and 60S ribosomal subunits) needed for translation of associated mRNAs,^61^ which could include BCL11A. Molecular characterization of IGF2BP1-containing RNPs and embodied mRNAs will be required to address this possibility.

Data from previous studies^39^ and those provided herein demonstrate that IGF2BP1-mediated reprogramming of hemoglobin expression in erythroblasts is achieved by a simultaneous increase in γ-globin and decrease in β-globin mRNA. Our 3C analysis revealed that IGF2BP1 restores long-range interactions between the LCR and γ-globin genes. There was, however, no appreciable effect on proximity of the LCR to the β-globin gene even in the situation where γ-globin mRNA made up two-thirds of total γ+β mRNA (Figure S5). A change in the LCR-β-globin interaction frequency has been varyingly observed upon induction of γ-globin transcripts.^49^^,^^50^ Repositioning of the LCR may be explained by reduction in BCL11A protein.^36^ However, it is intriguing to speculate that IGF2BP1 may interact with components of the LDB1/GATA-1/TAL1/FOG1/LMO2-containing complex or other multi-protein complexes involved in β-globin chromatin looping. This possibility can be addressed by targeted identification of proteins that immunoprecipitate with IGF2BP1 or by characterizing global changes in protein expression in adult erythroblasts reprogrammed to express HbF.

The benefit of HbF on severity of β-thalassemia and SCD is well documented. As a result, decades of research have been devoted to understanding the factors and mechanisms controlling the switch from HbF to HbA during development. Identification of proteins and noncoding RNAs involved in this process offers an opportunity to develop novel approaches to reactivate HbF.^41^ This can be accomplished by downregulating repressors or expressing activators of the endogenous γ-globin genes, respectively, using gene delivery and/or gene editing techniques. We have shown that lentivirus-mediated expression of IGF2BP1 is sufficient to cause potent reversal of adult hemoglobin production to the fetal type in erythroblasts derived from transduced CD34^+^ cells of patients with β-thalassemia or SCD. Reprogramming of high levels of hemoglobin expression is achieved at the transcriptional level by increased γ-globin combined with decreased β-globin mRNA, which shifts the total β-like globin mRNA profile to favor γ-globin. IGF2BP1 expression was predominantly coordinated with pancellular expression of HbF. That said, we consistently detected a small population of IGF2BP1-positive cells that lacked HbF (Figure 2C). The reason for this is currently unknown. IGF2BP1-dependent induction of HbF ameliorated the chain imbalance that occurs in β-thalassemia or potently suppressed expression of β^S^-globin in SCD. This is a critical point for SCD where γ-globin must compete with β^S^-globin for α-globin to yield beneficial HbF tetramers. Based on these data, it is possible that erythroid-specific expression of IGF2BP1 could provide dominant and curative levels of HbF in children or adults with β-thalassemia or SCD. Transplantation studies performed using healthy mice and models of β-thalassemia or SCD will be required to rigorously test the utility and safety of IGF2BP1 as a new therapeutic avenue for patients with severe β-hemoglobin disorders.

## Materials and Methods

### Lentiviral Vector Construction and Production

#### SPTA1-IGF2BP1-IRES-Puromycin (SIiP)

Details regarding construction of the self-inactivating (SIN) lentiviral vector (pLVX; Clontech Laboratories, Mountain View, CA, USA) encoding for bicistronic expression of the human IGF2BP1 cDNA (1,724 bp) and a puromycin resistance gene under transcriptional control of the human SPTA1 promoter have been reported.^39^

#### SPTA1-IGF2BP1-2A-Puromycin (SI2AP)

A 248-bp gBlock fragment was synthesized by IDT (Coralville, IA, USA) that included the 3′ end of IGF2BP1 cDNA (beginning at a unique *Bst*XI site and minus the stop codon) contiguous with the 2A peptide and puromycin gene sequences up to the *Xma*I site on the 5′ end of this gene. The fragment was provided in a cloning vector, excised with *Bst*XI and *Xma*I, and inserted between the same sites of SIiP to replace the IRES.

#### SPTA1-IGF2BP1-2A-ZsGreen (SI2AZG)

An 892-bp gBlock fragment was synthesized by IDT that again included IGF2BP1 sequences from the *Bst*XI contiguous with the 2A peptide and complete coding sequence for the ZsGreen fluorescent reporter (Clontech Laboratories, Mountain View, CA, USA). The cloning vector containing this fragment was digested with *Bst*XI and *Mlu*I and ligated with SIiP cut with the same enzymes to replace the IRES and puromycin resistance gene.

#### SFFV-IGF2BP1-2A-Puromycin (FI2AP)

The erythroid-specific SPTA1 promoter was replaced with the constitutive spleen focus-forming virus (SFFV) promoter. The SFFV promoter was PCR amplified from plasmid CL20-SFFV^62^ (a gift from Brian Sorrentino) with the addition of 5′-*Cla*I and 3′-*Xho*I restriction sites, and inserted into the pCR2.1 cloning vector (Invitrogen, Carlsbad, CA, USA). A 404-bp fragment was excised from a sequence-verified clone with *Cla*I and *Xho*I and inserted into SIiP cut with the same enzymes to remove the SPTA1 regulatory element.

Lentiviral vector particles pseudotyped with vesicular stomatitis virus glycoprotein (VSV-g) were prepared using human embryonic kidney 293T/17 cells (ATCC, Manassas, VA, USA; CRL-11268) and a four-plasmid transient transfection system.^63^ Viral supernatants were concentrated 10- to 20-fold by centrifugation at 20,000 rpm for 90 min at 4°C under vacuum and stored as aliquots at −80°C. Viral titer was determined by overnight transduction of 1 × 10^5^ K562 human erythroleukemia cells (ATCC; CCL-243) with serial dilutions of thawed virus in the presence of 8 μg/mL Polybrene. Flow cytometry was used to determine the percentage of cells that expressed ZsGreen on day 7 post-transduction or survived puromycin selection (propidium iodide negative fraction), which began 2 days after transduction and continued for 5 days, to calculate transducing units (TU) per mL of lentivirus for CD34^+^ cell transductions.

### CD34^+^ Cell Transduction and Erythroid Culture

Cytokine-mobilized peripheral blood or BM cells from healthy adult donors or patients with β-thalassemia major or SCD were collected according to protocols approved by the Institutional Review Board of St. Jude Children’s Research Hospital. CD34^+^ cells were recovered by positive selection using immunomagnetic beads, and cryopreserved cell products were obtained without personal identifiers. Purity and viability of processed cells was ≥90% for healthy donors and ≥80% for β-thalassemia or SCD patients. Purified CD34^+^ cells were also purchased from Lonza (Walkersville, MD, USA) or the Yale Cooperative Center of Excellence in Hematology (Yale School of Medicine, New Haven, CT, USA) to make comparisons between fetal and adult sources, respectively.

CD34^+^ cells were first cultured for 48 h in expansion medium consisting of StemSpan SFEM II (STEMCELL Technologies, Vancouver, BC, Canada) containing 2 U/mL human recombinant erythropoietin (EPO), 10 ng/mL stem cell factor (SCF), 1 ng/mL human recombinant interleukin-3 (IL-3), and 1 μM each dexamethasone and β-estradiol. Cells were transferred to RetroNectin-coated plates (Clontech Laboratories, Mountain View, CA, USA) and transduced with lentiviral particles (MOI of 10–20). After 48 h of exposure, transduced cells were enriched by FACS or selected for drug resistance by adding a low concentration of puromycin (0.5 μg/mL) to the culture medium. Enriched cell populations were cultured for 7 days at a concentration of 1–2 × 10^5^ cells/mL before they were collected for isolation of RNA (5 × 10^5^ cells) or protein (2 × 10^6^ cells). The remaining cells (ranging from 10 to 30 million) were transferred to differentiation medium (StemSpan SFEM II, 4 U/mL human recombinant EPO, and 0.5 mg/mL holo-transferrin) and cultured for 4–7 days at a concentration of 5 million cells/mL. Differentiated cells were lysed and hemoglobin tetramers evaluated by cellulose acetate hemoglobin electrophoresis and/or HPLC.

### qPCR

RNA was extracted (PureLink RNA mini; Life Technologies, Carlsbad, CA, USA) from cells 7 days after transduction, quantitated by NanoDrop, and 400 ng was used for first-strand cDNA synthesis (VILO with DNase; Invitrogen, Carlsbad, CA, USA). cDNAs were diluted to 100 ng/μL and 200 ng/reaction was used to detect IGF2BP1 (Hs00198023_m1), γ-globin(Hs00361131_g1), β-globin (Hs00747223_g1), BCL11A (Hs00256254_m1), LRF/ZBTB7A (Hs00792219_m1), and RNaseP (internal control, 4403328). TaqMan primer-probe sets were from Applied Biosystems. Data are expressed as percentage of RNaseP levels.

### Western Immunoblot

Whole-cell lysates, prepared with M-PER (mammalian protein extraction reagent) supplemented with 1× HALT protease inhibitors (Thermo Fisher Scientific, Waltham, MA, USA), were separated by SDS-PAGE and transferred to polyvinylidene fluoride (PVDF) membranes (Immobilon-P, Millipore). Signals were detected with SuperSignal West Pico (Pierce, Thermo Fisher Scientific, Waltham, MA, USA) and captured with a CCD (charge-coupled device) camera (Syngene, Frederick, MA, USA). Primary antibodies used were as follows: IGF2BP1 (Cell Signaling Technology, Danvers, MA, USA; clone D33A2), BCL11A (Abcam, Eugene, OR, USA; clone 14B5), GAPDH (Sigma-Aldrich, St. Louis, MO, USA; clone GAPDH-71.1) and α-tubulin (Active Motif, Carlsbad, CA, USA; clone 5-8-1-2). Horseradish peroxidase (HRP)-conjugated secondary antibodies (goat anti-mouse and goat anti-rabbit) were from Thermo Fisher Scientific.

### Flow Cytometry

Erythroblasts (1 × 10^5^ per condition) were treated with a Fix & Perm cell permeabilization kit (Invitrogen) according to the manufacturer’s instructions. Staining was performed during the permeabilization step. IGF2BP1 was detected with a monoclonal antibody (Cell Signaling Technology; clone D33A2, 1:100 dilution) and then Alexa Fluor 647-conjugated goat anti-rabbit secondary antibody (Life Technologies; 1:2,000 dilution). Cells treated with secondary antibody alone were used as a control. HbF was detected using phycoerythrin (PE)-conjugated mouse anti-human fetal hemoglobin monoclonal antibody (BD Pharmingen, San Jose, CA, USA; clone 2D12, 1:500 dilution); PE-conjugated mouse immunoglobulin G (IgG) (BD Pharmingen; 1:500 dilution) served as a control. Data were collected using the FACSAria II flow cytometer (BD Biosciences, San Jose, CA, USA) and analyzed with FlowJo v10.0 software. The percentage positive cells and geometric mean fluorescence intensity (MFI) was determined for each condition. In selected experiments, cells were simultaneously stained with antibodies to both IGF2BP1 and HbF and the percentage of double-labeled cells was determined.

### Hemoglobin Analysis

Differentiated erythroblasts (10–20 million) were lysed in hemolysate reagent (Helena Laboratories, Beaumont, TX, USA; 20 μL per 10 million cells) and refrigerated overnight. Supernatants were cleared of debris by centrifugation (14,000 rpm at 4°C for 15 min), and hemoglobin tetramers were identified by cellulose acetate electrophoresis as described^17^ and HPLC using a G7 analyzer (Tosoh Bioscience, San Francisco, CA, USA) and β-thalassemia settings. Globin chain expression was performed for selected samples on a TSP Specta HPLC system using a LiChristopher 100 RP-8 column and a gradient of acetonitrile-methanol-sodium chloride.^64^ Controls included a standard with known amounts of adult (HbA and HbA_2_), fetal (HbF), and sickle (HbS) hemoglobin (AFSA2 Hemo Control, Helena Laboratories), as well as hemolysates prepared from adult peripheral blood or umbilical cord blood samples.

### 3C Assay

The 3C assay was performed as described^48^ with minor modifications. CD34^+^ cells from a healthy adult donor were transduced with control or IGF2BP1 lentivirus in triplicate. Culture-differentiated erythroblasts (8–9 million per condition) were collected 8 days after transduction and fixed with 1% formaldehyde. Isolated nuclei were digested overnight with *Eco*RI and ligated for 4 h. Interaction frequency between the anchor fragment and regions of interest in the β-globin locus were determined by SYBR Green quantitative real-time PCR using published primers.^49^ Interaction frequency between two fragments within the α-tubulin gene was used for the internal normalization control. Interaction frequency for the fragment containing the ^G^γ-globin gene is representative of both the ^A^γ- and ^G^γ-globin genes, as these sequences cannot be effectively discriminated due to high sequence homology.^49^

### Statistical Analysis

Microsoft Excel or GraphPad Prism 5 was used to determine descriptive statistics (mean ± SD or SEM), and significant differences between mean values were determined by an unpaired Student’s t test (two-tailed) or one-way ANOVA with a Newman-Keuls *post hoc* test. Values of p are indicated by asterisks in the figures with level of significance reported.

## Author Contributions

C.B.C. performed the research, analyzed data, and wrote the manuscript. J.G., K.P., C.B., and Y.T.L. contributed to performing the research. D.L. performed reverse-phase HPLC and edited the manuscript. X.G. and A.D. performed 3C experiments and edited the manuscript. J.L.M. provided critical reagents, technical advice, and edited the manuscript. A.W. designed the research, analyzed data, wrote the manuscript, and was responsible for organization of the research effort. All authors read and approved the final manuscript.

## Conflicts of Interest

The authors declare no competing interests.

## Supplemental Information

Document S1. Figures S1–S5

Document S2. Article plus Supplemental Information
