# Supplementary material for: The mRNA-Binding Protein IGF2BP1 Restores Fetal Hemoglobin in Cultured Erythroid Cells from Patients with β-Hemoglobin Disorders
Source: Mol Ther Methods Clin Dev. 2020 Jan 31;17:429–40. doi: 10.1016/j.omtm.2020.01.011 (PMC7056608; doi:10.1016/j.omtm.2020.01.011)
Supplement: Document S1. Figures S1–S5 [file mmc1.pdf]

OMTM, Volume *17*

## Supplemental Information

# The mRNA-Binding Protein IGF2BP1 Restores Fetal Hemoglobin in Cultured Erythroid Cells from Patients with β-Hemoglobin Disorders

**Christopher B. Chambers, Jeffrey Gross, Katherine Pratt, Xiang Guo, Colleen Byrnes, Y. Terry Lee, Donald Lavelle, Ann Dean, Jeffery L. Miller, and Andrew Wilber**

Supplementary Materials for

# The mRNA-binding protein IGF2BP1 restores fetal hemoglobin in cultured erythroid cells from patients with β-hemoglobin disorders

Christopher B. Chambers, Jeffrey Gross, Katherine Pratt, Xiang Guo, Colleen Byrnes, Y. Terry Lee, Donald Lavelle, Ann Dean, Jeffery L. Miller, and Andrew Wilber*

* To whom correspondence should be addressed. E-mail: awilber@siumed.edu

This supplementary file includes:

Supplementary Figures. S1-S5

Figure S1.

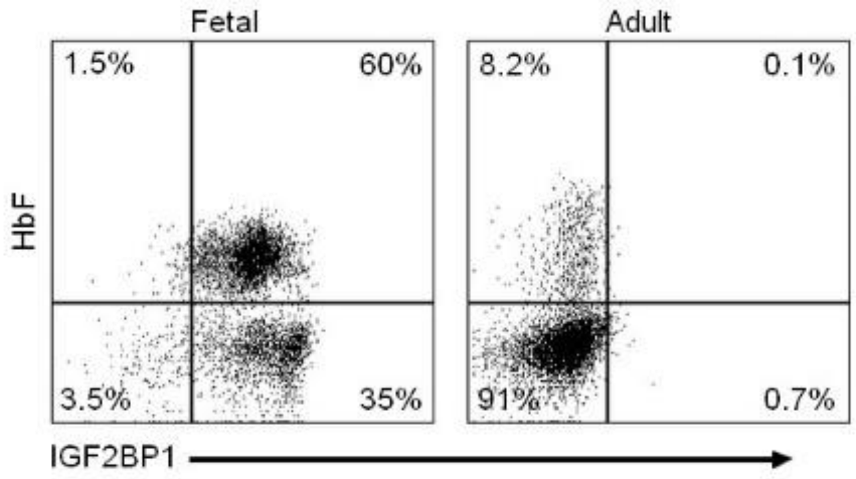


**Figure S1. Expression of IGF2BP1 and HbF in fetal and adult erythroblasts.** Flow cytometry dot plots showing expression of IGF2BP1 and fetal hemoglobin (HbF) for erythroblasts derived from fetal liver (fetal, left) or adult bone marrow (adult, right) $CD34^+$ cells. Percentage of positive cells is indicated in each quadrant.

Figure S2.

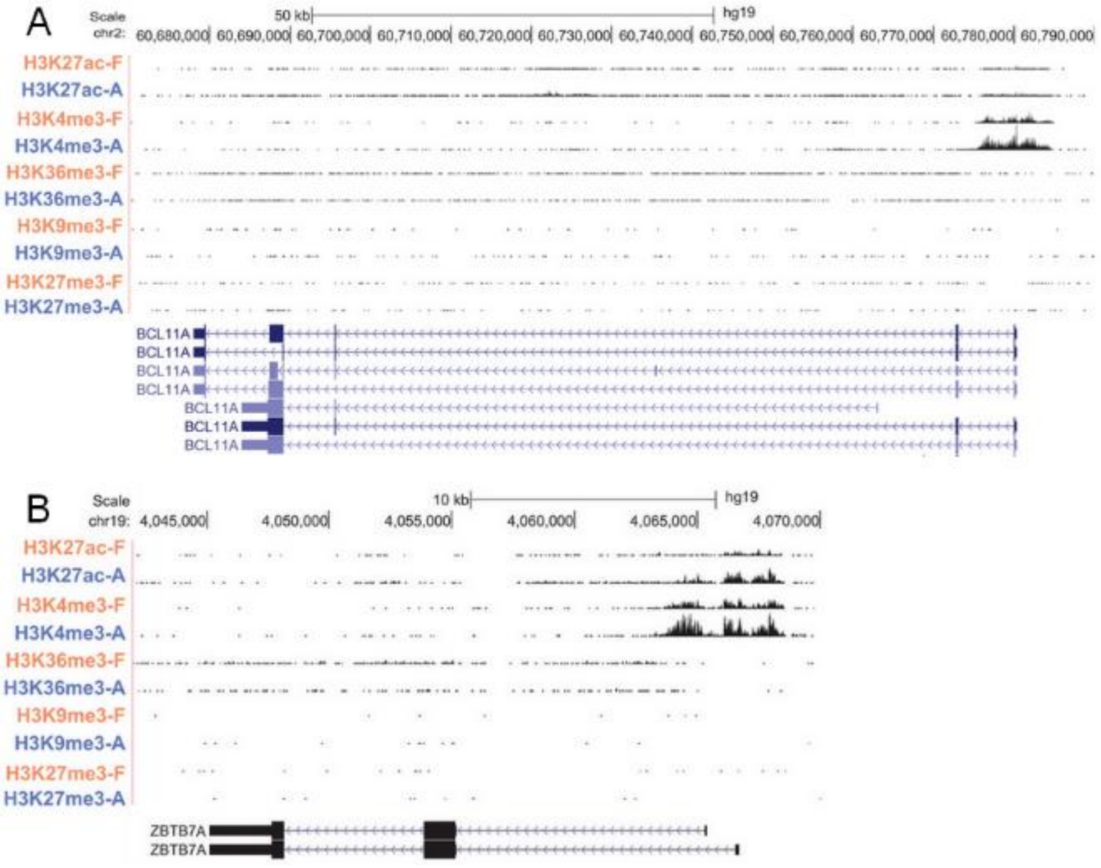


**Figure S2. Epigenetic analysis of BCL11A and LRF/ZBTB7A in fetal and adult erythroblasts.** ChIP-sequencing results for culture-differentiated fetal and adult erythroblasts (37) were re-analyzed to determine epigenetic modifications for (**A**) BCL11A and (**B**) LRF/ZBTB7A. Shown are results for markers of active chromatin: H3K27 acetylation (H3K27ac), H3K4 trimethylation (H3K4me3), and H3K36 trimethylation (H3K36me3) or repressive chromatin: H3K9 trimethylation (H3K9me3) and H3K27 trimethylation (H3K27me3) in fetal (orange) and adult cells (blue).

Figure S3.

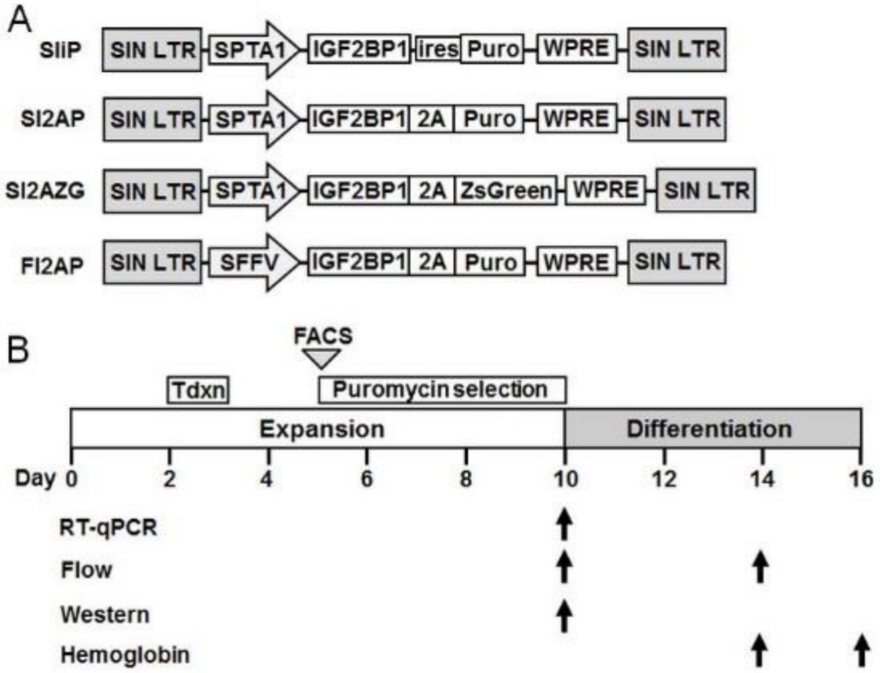


**Figure S3. IGF2BP1 lentiviral vectors and experimental conditions.** (**A**) Integrated proviral form of the pLVX (Clontech Laboratories) self-inactivating (SIN) lentiviral vectors encoding for expression of IGF2BP1 and puromycin or ZsGreen under transcriptional control of the erythroid-specific human spectrin alpha gene (SPTA1) promoter or constitutive spleen focus forming virus (SFFV) promoter/enhancer. The positions of the internal ribosome entry site (ires), porcine teschovirus-1 2A peptide (2A), and woodchuck post-transcriptional regulatory element (WPRE) are shown. (**B**) Experimental schema with time course of expansion and differentiation phases indicated. Identified are intervals for viral transduction (Tdxn) and puromycin selection or time points for FACS and each experimental determination.

Figure S4.

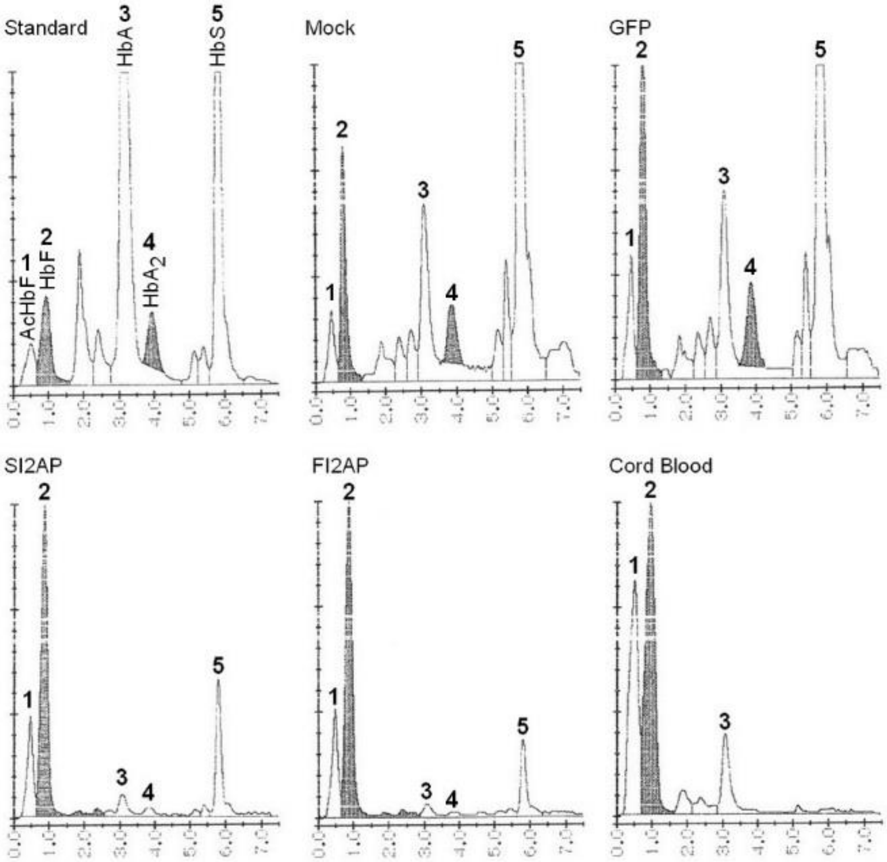


**Figure S4. HPLC analysis of hemolysates from SCD erythroblast expressing IGF2BP1.** Steady-state bone marrow CD34+ cells from a patient with SCD were mock treated or transduced lentivirus particles encoding for expression of GFP (control) or IGF2BP1 under control of the SPTA1 (SI2AP) or SFFV (FI2AP) promoter. HPLC trace files for hemolysates from differentiated cells were obtained using β-thalassemia program settings, which automatically shades peaks for HbF and $HbA_2$. A standard with known concentrations of fetal (HbF), adult (HbA, $HbA_2$) and sickle (HbS) hemoglobin was used to identify peaks, which are labeled and numbered. A cord blood sample, which does not contain $HbA_2$ or HbS, was also included as an additional control.

Figure S5.

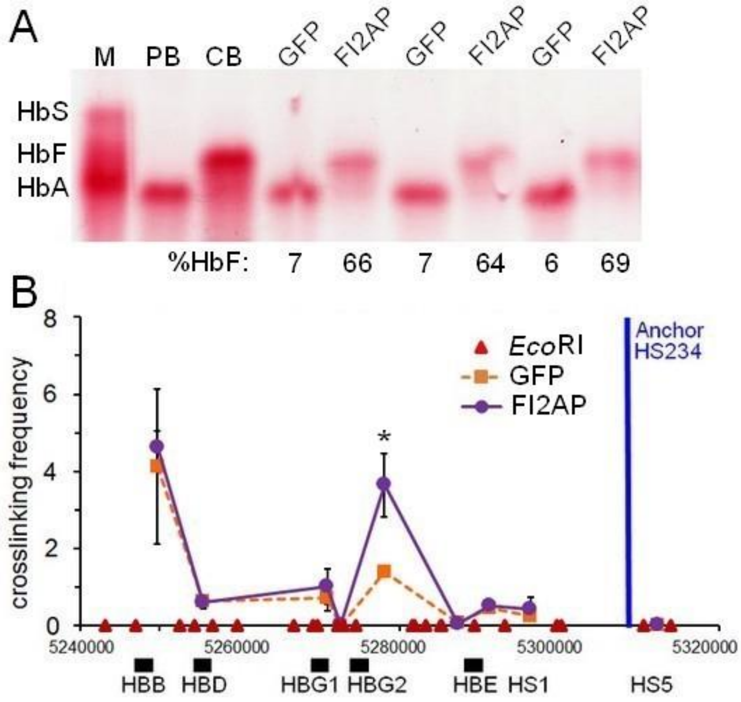


**Figure S5. LCR interaction with γ-globin genes is enriched in adult erythroblasts expressing IGF2BP1.** Cytokine-mobilized $CD34^+$ cells from a healthy adult donor were transduced in triplicate with GFP control or FI2AP lentivirus particles. (**A**) Hemoglobin electrophoresis of differentiated cell lysates. Percentage HbF of total hemoglobin [HbF + HbS] determined by HPLC is reported below each lane. Control samples were from adult peripheral blood (PB) or umbilical cord blood (CB). M, standard consisting of sickle (HbS), fetal (HbF) and adult (HbA) hemoglobin. (**B**) 3C assay measuring relative crosslinking frequencies between the anchor fragment (vertical red line) and globin genes in control (orange) and IGF2BP1 (purple) expressing cells. Each *Eco*RI cleavage site is represented by a red triangle, and globin genes identified with black rectangles. Data are plotted as mean ± SD. *, $p \leq 0.01$ determined by unpaired Student's *t*-test (two-tailed).
